# A Hybrid Lifetime Extended Directional Approach for WBANs

**DOI:** 10.3390/s151128005

**Published:** 2015-11-05

**Authors:** Changle Li, Xiaoming Yuan, Li Yang, Yueyang Song

**Affiliations:** State Key Laboratory of Integrated Services Networks, Xidian University, Xi’an 710071, China; E-Mails: xmyuan2013@163.com (X.Y.); yangli_jju@163.com (L.Y.); yueyangsong@foxmail.com (Y.S.)

**Keywords:** WBAN, MAC, directional antenna, energy efficiency, IEEE 802.15.6

## Abstract

Wireless Body Area Networks (WBANs) can provide real-time and reliable health monitoring, attributing to the human-centered and sensor interoperability properties. WBANs have become a key component of the ubiquitous eHealth (electronic health) revolution that prospers on the basis of information and communication technologies. The prime consideration in WBAN is how to maximize the network lifetime with battery-powered sensor nodes in energy constraint. Novel solutions in Medium Access Control (MAC) protocols are imperative to satisfy the particular BAN scenario and the need of excellent energy efficiency in healthcare applications. In this paper, we propose a hybrid Lifetime Extended Directional Approach (LEDA) MAC protocol based on IEEE 802.15.6 to reduce energy consumption and prolong network lifetime. The LEDA MAC protocol takes full advantages of directional superiority in energy saving that employs multi-beam directional mode in Carrier Sense Multiple Access/Collision Avoidance (CSMA/CA) and single-beam directional mode in Time Division Multiple Access (TDMA) for alternative in data reservation and transmission according to the traffic varieties. Moreover, the impacts of some inherent problems of directional antennas such as deafness and hidden terminal problem can be decreased owing to that all nodes generate individual beam according to user priorities designated. Furthermore, LEDA MAC employs a Dynamic Polled Allocation Period (DPAP) for burst data transmissions to increase the network reliability and adaptability. Extensive analysis and simulation results show that the proposed LEDA MAC protocol achieves extended network lifetime with improved performance compared with IEEE 802.15.6.

## 1. Introduction

In Wireless Body Area Network (WBAN), sensors are attached to or implanted in human body to monitor physiological, behavioral and other health-related information and propagate their readings back to hub or the base station for diagnosis and prescription. A WBAN can provide continuous and reliable health monitoring for human body with great freedom and comfort, regardless of the limitation of living in or near the hospital and healthcare facility, changing the paradigm of healthcare systems. Besides, the WBAN can also support better user experience, leisure and entertainment applications besides medical applications. It follows that WBAN is flourishing in eHealth and popular among patients and healthy people, owing to the ubiquitous networking functionalities for various application scenarios [1]. Moreover, the WBAN is also engaged in the research on machine-to-machine systems for mHealth (mobile health) [2] from communication perspective.

There are many emerging challenges [3,4,5] in the communication of sensors and hub in WBAN. The main limitations are provided by the energy-constraint wearable and implanted sensor devices and by the fact that the emergency vital signs should be transmitted in reliability and timeliness. The survival time of wearable and implanted sensor devices that mostly rely on batteries as energy supplies is anticipated to progress to at least a few months or years, especially for the implanted sensor nodes. Ascribing the difficulty in battery replacement or recharge, sensor nodes in WBAN must be highly provident in energy consumption with energy-efficient communication mechanisms. The importance of reliability and timeliness is distinct because the successful and abortive transmission of emergency data in time literally means the difference between life and death. Besides, disparate sensor nodes are different in traffic varieties such as information, images or video that the wide range of arrival rates lead to strong interferences among sensors nodes interoperate with the exclusive hub. The orientation of nodes with reference to each other and human body can also attenuate the strength of electromagnetic waves in WBAN. As a corollary, energy-efficient and reliable Medium Access Control (MAC) protocols in accurate propagation without interference are successful solutions to the challenges.

Numerous studies are dedicated to the development of various MAC protocols to increase network reliability and energy utilization for Wireless Sensor Network (WSN) or Wireless Personal Area Network (WPAN). As a special wireless sensor network, WBAN has distinctive characteristics from WSN and WPAN. Because WBAN is human-centered with heterogeneous sensor nodes in different traffic varieties while WSN considers homogeneous sensors without taking human body into account and WPAN most concentrates on the interaction of personal communication devices. WSN or WPAN cannot afford specific solutions to communication and application requirements in WBAN [6]. Besides, most of current MAC protocols are designed to adopt omnidirectional antennas, which cannot benefit for energy consumption and accurate propagation. Whereas, directional antenna can transmit and receive data only in one or several specific directions in focused beam without interference to other nodes and substantially reduce energy waste. What is more, directional antenna can increase the channel utilization that simultaneous transmissions are allowed in the multi-beam directional antenna mode. To maintain the same link quality, the directional antennas are capable to consume less energy and extend the network lifetime. Directional antenna can also achieve the communication range extension compared with omni-directional antennas in the same energy consumption condition. High energy efficiency and transmission reliability of directional antenna technology supplies more optional choices in wireless networks, especially in battery-operated networks.

Using directional antennas on designing MAC protocols in wireless networks is investigated by Choudhury, R.R. *et al.* in [7]. The application of directional antennas in ad hoc networks has been highly appreciated to increase spatial reuse, improve transmission reliability and save the power consumption. A survey with qualitative comparison of MAC protocols with beamforming antennas is presented in [8]. The challenges in the design of directional MAC protocols have been introduced in [8] as well. Recently, increasing attentions have been focused on the directional MAC devising in WBAN on account of the high-energy efficiency, no interference, and exact propagation of directional antenna. Besides, the location of sensor nodes is relatively fixed in WBAN and the movement range of human body is small in most situations when people are desired to check healthy data records. All the aforementioned facilitates the application of directional antennas in WBAN.

In this paper, we propose a hybrid Lifetime Extended Directional Approach (LEDA) MAC protocol for energy-efficient and reliable data transmissions on the basis of IEEE 802.15.6 [9]. IEEE 802.15.6 is an international standard for short-range, low power consumption wireless communication especially for WBANs. It provides the technical supports for Physical Layer (PHY) and MAC layer technology. The customary monitoring data in WBAN are periodically. Scheduled-based MAC protocols provide good solutions to the data transmission demands. Directional antenna technology and scheduled access mechanism are integrated in LEDA MAC protocol to release the burden of energy consumption, which can largely extend the nodes’ lifetime and guarantee the reliability of periodic data transmissions. Besides, two directional modes are designed for slots reservation and data transmission. The directional antenna, by switching between multi-beam and single-beam mode, can extremely reduce energy requirements and solve the conventional deafness and hidden terminal problems [10,11]. To some extent, on-demand traffic and some emergency data are burst traffic. Considering burst data are mostly critical in severe condition, a Dynamic Polled Allocation Period (DPAP) is allocated to accommodate the timeliness principle, decreasing the average packet delay. If there were no burst data, there would be no DPAP. Accordingly, the superframe of LEDA MAC is modified.

The performance evaluation has been conducted in terms of the throughput, delay, energy consumption and network lifetime theoretical analysis. The simulation results also consolidate the theoretical analysis and show the fact that LEDA MAC can significantly extend network lifetime with improved performance. The key contributions of our study are summarized as follows.

(1) Directional antenna technology is employed in LEDA MAC for sensor nodes in different sectors. The LEDA MAC switches between multi-beam and single-beam directional mode for data reservation and transmission. The proposed single-beam transmission mode enjoys features of simplicity in system configuration, low complexity in data processing. What is more, the adaptive design in LEDA MAC avoids the impacts of deafness and hidden terminal problem, decreases the energy waste and thus extends the network lifetime.
(2) The hybrid MAC combines the strength of contended and scheduled mechanism. Data request frames for reservation access the channel in contention-based mechanism and packet transmissions are in scheduled mechanism, achieving high link utilization and low packet delay in unsaturated network and reducing collision probability in saturated network. Both the data reservation and transmission are according to the User Priorities (UP), ensuring the privilege of emergency data.
(3) Considering burst data, a modified adaptive superframe is proposed. A dynamic polled allocation period is designed to guarantee the on-demand and emergency data transmissions. The specific fields of frame control frame for polled allocation are supplied, which facilitates the study for other researchers.

The rest paper is organized as follows. In Section 2, we discuss the related work. Section 3 presents the specific LEDA MAC protocol. In section 4, we derive analytical expressions for the performance of LEDA MAC protocol. Section 5 discusses the analysis and simulation results. Finally, Section 6 concludes the paper and highlights our future work.

## 2. Related Work

### 2.1. Previous Work

In general, MAC protocols are designed to extend the network lifetime by reducing the energy waste in packet collision, idle listening, overhearing, and control packets overhead. A survey of low-power MAC protocols is studied in [12]. In general, energy-efficient MAC protocols are contention based, scheduled based or hybrid based access mechanisms. S-MAC [13], T-MAC [14] are typical contention-based MAC protocols that try to solve idle listening by employing a synchronized schedule between the sensor nodes. The performance of IEEE 802.15.6 Carrier Sense Multiple Access/Collision Avoidance (CSMA/CA) protocol in different frequency bands and data rates is studied in [15,16,17,18]. A cooperative energy harvesting MAC is proposed in [19] following CSMA/CA protocol rules that allows relay nodes to charge their batteries. However, sometimes battery recharge is difficult, and these contention-based protocols suffer high collision probability with the increasing data arrival rate and the number of sensor nodes, leading to more energy consumption. At the same time, idle listening wastes a lot of energy. Scheduled based MAC protocols in [20,21,22,23,24,25,26,27] supply better solutions to the traffic related problems since there is no need to concern the contention and idle listening problems. A study of ultra-low power Time Division Multiple Access (TDMA) MAC protocols based on IEEE 802.15.4 is presented in [22]. References [23,24,25] pay attention to wakeup mechanism for different traffic varieties. A cross-layer based battery-aware TDMA MAC [26] is designed to prolong battery lifespan. However, these protocols are IEEE 802.15.4 based that pay attention to the low rate WPAN and concentrate on the communication between different individual and household devices. Furthermore, they do not take the user priorities into consideration, leading the medical business especially the emergency traffic making no sense in limited time. S-TDMA [27] on the basis of IEEE 802.15.6 employs both TDMA and unified scheduling scheme for energy-efficiency. Nevertheless, the slots allocation does not ponder the traffic varieties in different user priorities. The hybrid protocols such as [28,29,30,31,32,33,34] combines the advantages of TDMA and CSMA mechanisms achieving high channel utilization. Reference [30] divides the superframe into downlink and uplink frame, and the uplink frame is further subdivided into a contention access period and contention free period. An energy-aware hybrid access scheme named HEH-BMAC exploits energy harvesting at sensor nodes has been presented in [31]. The HEH-BMAC considers energy level of sensor nodes that a reserved polling access phase is for nodes with high energy level while a random access phase is for nodes with low energy level. The design that request frames reservation in CSMA and data transmission in TDMA in [32,33] satisfies the diverse demands for different nodes in WBAN, and more adaptive under both low and high contention scenarios. To complement the stringent Quality of Service (QoS) demands, a QoS-aware energy management scheme namely PEH-QoS is introduced in [34]. The PEH-QoS control scheme uses the hybrid IEEE 802.15.6 MAC to increase the throughput and energy efficiency for WBAN with considering different human activities, *i.e.*, relaxing, walking, running and cycling. The hybrid based MAC protocols provide reliable propagations and can be more adaptive in slots allocation for heterogeneous data with different priorities.

However, most of the aforementioned MAC protocols are designed in omni-directional antenna, leading to unnecessary energy consumption and interference to other nodes. There is no need to worry about these problems when we involve directional antenna technology into MAC protocol design. Directional antenna can provide some interesting advantages. The coverage pattern is petal-shaped in lower transmit power, which can shield the interference signal from neighbor nodes, increasing the resistance to interference. The directional beams focus the receivers in specific directions reducing the unnecessary receipts by the undesirable nodes within the transmission range, obtaining better performance in energy conservation. Some kinds of antennas are designed in [35,36,37] for Ultra-WBAN applications. The performances of radiation efficiency and Specific Absorption Rate (SAR) are studied. A cooperative beamforming scheme B-MIMO [38] in scheduling strategy is envisaged for WBAN in terms of energy efficiency and block error rate. However, extra communication overhead cannot be neglected. Directional MAC [39] uses TDMA and multi beam antenna approach to increase network capacity. Whereas, the performance of the directional MAC has not been evaluated. The multi-beam adaptive arrays integrated with slotted aloha directional MAC protocol for WBAN is presented in [40]. Multiple communications can be initiated by different nodes concurrently instead of only one transmission in omni-directional mode. The authors aim to decrease the delay of accessing the hub, and simultaneous transmissions in case emergency. However, sometimes, simultaneous transmissions may not be appropriate for urgent situations where uninterrupted communications generate from more than one node. This would be more complexity in data processing. At the same time, quite complex antenna arrays system are needed by hub for heterogeneous data arrival rate of different nodes. A quite effective and simple system is more appropriate for WBAN.

Our proposed LEDA MAC protocol employs hybrid based access mechanism with directional antennas on development of IEEE 802.15.6. Our motivation is minimizing the energy consumption to extend nodes’ lifetime, minimizing idle listening, overheads and collision, providing an efficient and timely response to emergency or burst data. They are accomplished through this: Nodes contend for the sequence number in Multi-beam Transmission Period (MTP) according to the UPs. Hub interoperate with nodes sequentially in single-beam mode according to the distributive allocation information. All nodes, including hub, turn to inactive or sleep mode if there are no data transmission. Dynamic polled allocation is designed in Single-beam Transmission Period (STP) for burst data improving the utilization of scheduled scheme. The elaborate conception that system transmits data in single-beam mode reduces the complexity of data processing that there is no simultaneous data pending to be managed and decreases the energy consumption more effectively than that of omni-directional antennas. The specific description will be presented in Section 3.

The proposed LEDA MAC is an improved work of the Energy-efficient Directional Approach Medium Access Control (EDA-MAC) in [41]. Compared with the EDA-MAC, the LEDA MAC introduces a system model and traffic transfer model for data transmission around human body. Specific fields of data request frame and control frame for polled allocation are illustrated. More performance indicators on average packet delay and network lifetime have been evaluated both in theoretical and simulation results. Furthermore, an experimental scenario is provided in this paper to share some interim achievements.

### 2.2. Preliminary Study

#### 2.2.1. Directional Antennas

**Figure 1:**
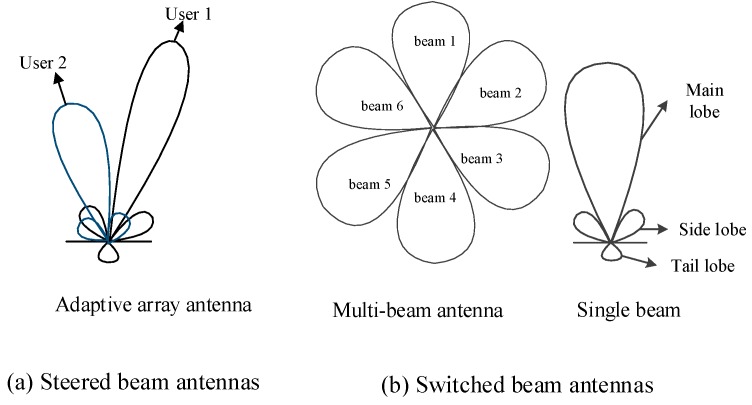
Two main kinds of directional antennas. (**a**) Steered beam antennas; (**b**) Switched beam antennas.

A directional antenna radiation pattern is usually composed of a main lobe with high gain and smaller gain side and tail lobes. The ideal directional antennas are supposed to have constant gain in the main lobe and zero outside. Directional antennas, equally as described as beam antennas, are divided into two main types: steered beam antennas and switched beam antennas. Steered beam antennas can also be referred to as adaptive array antennas, as shown in Figure 1a. Adaptive array antennas can focus the receiver in its main lobe by adjusting the weighted phase and amplitude of each antenna element, the direction of which changes as the user moves. What cannot be neglected of the system, is the added complexity of signal processing and the significant increase in energy consumption. In switched beam antennas, there are some fixed equal-sized sectors with covering 2π/*n* area around the antenna. Besides, switched beam antennas can be divided into two types: multi-beam and single-beam directional antennas. In multi-beam systems, every beam focuses a different user. Simultaneous transmissions are allowed at the same time and frequency. On the contrary, only one active beam is active in single beam systems at a given time. A 6-sector switched beam antenna is depicted in Figure 1b. Each sector includes a single beam. The switched beam antenna enjoys a small fraction of complexity and expense with energy saving.

The Friis transmission equation [42] is the most fundamental equations in antenna theory. It is used to calculate the transmitted and received power between two antennas in free space. Let *R* represents the distance between the transmitting antenna and the receiving antenna. *P_t_* represents the output power value of transmitting antenna to send data frames while *P_r_* denotes the available power at the receiving antenna terminals for data reception, and then we have

(1) $$P_{r}=P_{t}G_{t}G_{r}{\left(\frac{\lambda }{4\pi R}\right)}^{2}$$

where *G_t_* and *G_r_* represent the antenna gains of the transmitting and receiving antennas, respectively. Antenna gain in a specified direction is the ratio of the power required of a reference antenna to the power supplied of the given antenna to produce the same field strength at the same distance, and the gain is usually measured in dBi while the reference antenna is an omni-directional antenna. The inverse of the factor in parentheses is the free-space path loss and λ is the wavelength Equation (1) can be modified to Equation (2) when the power has units of dBm or dBW.

(2) $$P_{r}=P_{t}+G_{t}+G_{r}+20\log_{10}\left(\frac{\lambda }{4\pi R}\right)$$

### 2.3. IEEE 802.15.6

IEEE 802.15.6 [9] specifies MAC and PHY layer standard for short-range, low power consumption wireless communications for WBAN. What is more, IEEE 802.15.6 considers effects on portable antennas owing to human body factors (varying with sex, skin, weight, *etc.*); antenna radiation pattern shaping to minimize the SAR and related changes resulted from the user motions.

According to IEEE 802.15.6, hub establishes a time base, which divides the time axis into beacon periods (superframes) to support time reference allocations in its BAN. Each superframe is bounded by a beacon period of equal length allocation slots (maximum is 255). The IEEE 802.15.6 network operates in one of the following modes: the beacon mode with superframes, the non-beacon mode with superframes and the non-beacon mode without superframes.

**Figure 2:**
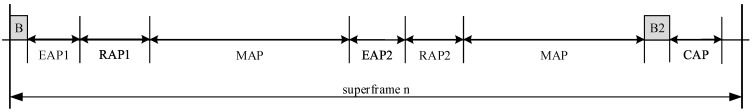
Layout of access phases in a superframe for beacon mode.

The beacon is transmitted by the hub at the start of each superframe to notify network management information, such as power management, clock synchronization and the super frame structure. The superframe structure of the beacon mode with superframes is shown in Figure 2, where B is short for beacon. Except for the beacon, the superframe is divided into seven access phases: Exclusive Access Phase1 (EAP1), EAP2, Random Accesses Phase1 (RAP1), RAP2, two Managed Access Phases (MAPs), and Contention Access Phase (CAP). In EAP, RAP and CAP periods, nodes contend for the resource allocation using either CSMA/CA or slotted Aloha access procedure. The EAP1 and EAP2 are only used for highest priority (totally eight defined UPs) traffic such as reporting emergency events while there is no traffic variety limits in RAP and CAP. The MAP supports scheduled access, unscheduled access and improvised access.

**Figure 3:**
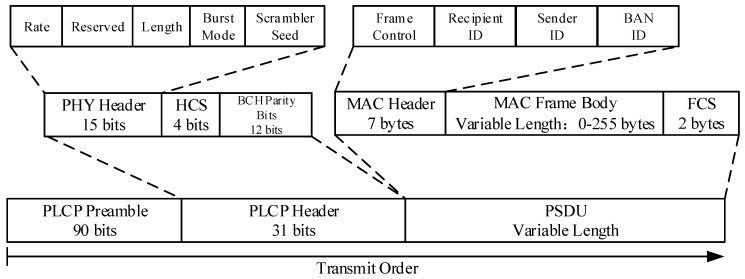
Physical layer protocol data unit structure of IEEE 802.15.6.

IEEE 802.15.6 supports Narrowband (NB), Ultra Wideband (UWB) and Human Body Communications (HBC) PHY. The NB PHY is responsible for activation and deactivation of the radio transceiver, Clear Channel Assessment (CCA) within the current channel even data transmission and reception. NB Physical Layer Protocol Data Unit (PPDU) consists of three components: The Physical Layer Convergence Protocol (PLCP) preamble, the PLCP header, and the physical-layer service data unit (PSDU). The structure of PPDU is illustrated in Figure 3. The IEEE 802.15.6 MAC sets the Burst Mode bit in PHY header to indicate whether the next packet is part of burst mode transmission.

## 3. The Proposed MAC Protocol

LEDA MAC protocol is a directional MAC designed to maximum the network lifetime as long as possible for health monitoring. The network lifetime is defined as the time duration from the start of network operation to the moment when any sensor node runs out of its energy. We classify monitoring data in healthcare into normal data and burst data. Usually, the sensor nodes initiate the data transmissions, so only uplink data transmissions are considered in our work.

### 3.1. System Model

**Figure 4:**
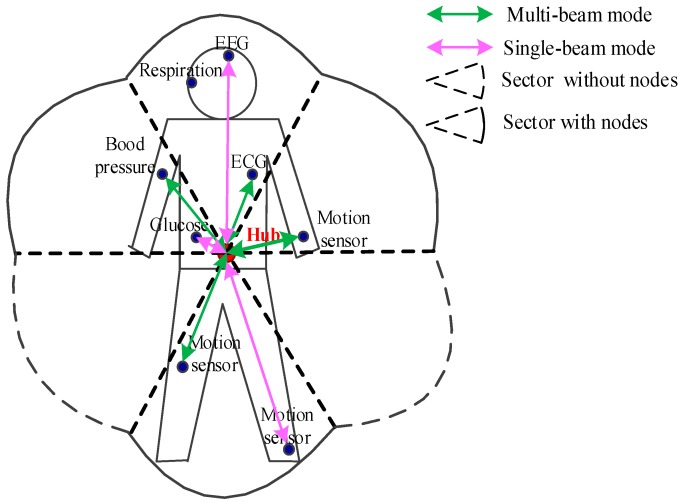
System model in wireless body area network.

An antenna model in WBAN is presented in Figure 4. In this model, hub and nodes are equipped with the switched beam antennas. The antenna system offers two modes of operation: multi-beam mode and single-beam mode. In multi-beam mode, the hub produces multiple beams to divide the space into several independent sectors to cover the human body. Simultaneous transmissions are allowed in the multi-beam mode. Beams are active only in sectors that have data pending to be transmitted. In single-beam mode, only one beam is active, thus effectively reducing the number of nodes involved in competition. Collisions only occur when more than one node has pending data to be sent in the same sector. If the relevant information of periodic monitoring data (location, UP, length, *etc.*) has been acquired by the hub, the hub can switch its beam to the node having data to transmit. Once there is no traffic in the network, all nodes turn into inactive to save energy.

### 3.2. LEDA MAC Protocol

#### 3.2.1. Superframe of LEDA MAC Protocol

**Figure 5:**
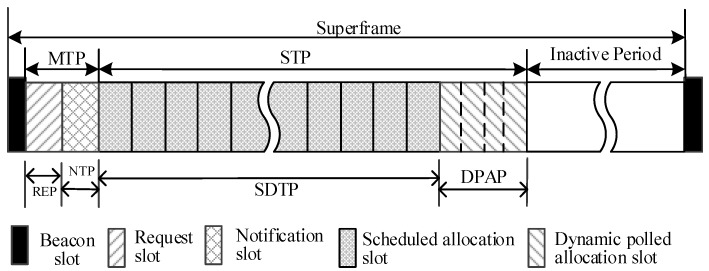
Modified superframe layout of lifetime extended directional approach medium access control.

The superframe of LEDA MAC is shown in Figure 5. There are mainly three periods in LEDA MAC protocol: Multi-beam Transmission Period (MTP), Single-beam Transmission Period (STP) and Inactive Period. The MTP and STP correspond to the RAP and MAP in IEEE 802.15.6, respectively.

**Figure 6:**
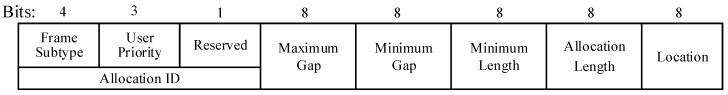
The Data request frame format.
  (c) Notification Period (NTP): Hub broadcasts Notification Frames (NTFs) in NTP to notify the assignment information. The related information about transmission order and the number of time slots needed to send data in NTFs are appointed by the hub according to the information included in the DRFs gathered in REP. A user with higher UP can be assigned slots preferentially. Prioritized access of differing UPs shall be attained through the predefined relationship in Table 1.

(1) Multi-Beam Transmission Period (MTP): All nodes work in multi-beam antenna mode at the start of a superframe. Simultaneous transmissions are permitted in multi-beam mode. The specific function of each phase in MTP is introduced in the following subsections.
  (a) Beacon: The beacon frame is transmitted by the hub at the start of each superframe to notify the network management of information, such as power management of the nodes in the WBAN, and to facilitate clock synchronization therein. All the nodes, the beams of which point to hub, can be informed.
  (b) Request Period (REP): Only Data Request Frames (DRFs) instead of data frames could be sent in REP in multi-beam mode. Data request frame always includes the user priority, the length of data packet, the location of nodes and any other information, as depicted in Figure 6. DRFs access the channel in CSMA/CA mechanism for slots reservation. The collision probability can be smaller because the request frame is generally short.

**Table 1:** User priorities mapping.

Priority	User Priority(UP)	Traffic Designation	Frame Type
Lowest	0	Background (BK)	Data
	1	Best effort (BE)	Data
	2	Excellent effort (EE)	Data
	3	Video (VI)	Data
	4	Voice (VO)	Data or management
	5	Medical data or network control	Data or management
	6	High priority medical data or network control	Data or management
Highest	7	Emergency or medical implanted event report	Data
(2) Single-Beam Transmission Period (STP): In LEDA MAC protocol, STP is divided into two parts: single-beam data transmission period and dynamic polled access period.
  (a) Single-Beam Data Transmission Period (SDTP): In SDTP, hub directionally interacts with nodes in each sector according to the notification packets. Any data transmissions out of node’s allocated slots will not be initiated. The data interaction in STP is generated in an active beam, which can reduce the interference of neighbor nodes. The nodes with no data transmission will turn into inactive state to save energy.
  (b) Dynamic Polled Allocation Period (DPAP): Burst traffic may emerge before the last frame transaction. If slots have not been reserved for burst data, the data will have to wait until the next superframe to be transmitted, which will lead high packet delay. To solve this problem, dynamic polled allocation period is designed in STP. Hub will send control type frame I-ACK+Poll to provide polled allocation. The fields of the MAC header are listed below in Figure 7.

In beacon mode with superframes, the value of Access Mode is zero. The More Data field is set to zero if via the current frame hub grants an immediate polled allocation that starts pSIFS thereafter and ends at the end of the allocation slot that is numbered *E* (Poll-Post Window value) in the current superframe for *N* = 0, or in the next *N*th superframe not counting the current one for *N* > 0. More Data fields are set to one if hub grants a future poll at the start of the numbered *S* allocation and located in the current superframe for *F* = 0, or in the next *F*th superframe not counting the current one for *F* > 0.

**Figure 7:**
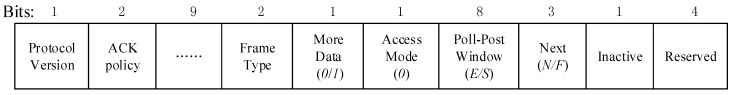
The fields of frame control format for polled allocation.

Node can send emergency or on-demand data in its new designated slots. If the traffic is heavy, hub cannot distribute enough slots for nodes, and then a future poll is sent to node to indicate that the newly arrived data should wait the next superframe. It is important to note that when all nodes have no burst packets, the polled access period will not exist. The length of DPAP is adaptive. When there are too many newly arrived data in the network, the length of DPAP is maximum and there is no inactive state period.

**Figure 8:**
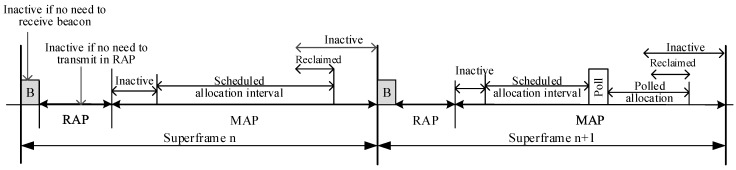
Inactive period in superframe.

(3) Inactive Period: Inactive state is an internal power management state that is not ready for frame transmission and reception. A node can turn to inactive state, *i.e.*, sleep mode over some time intervals in a superframe to save energy, if it does not need to transmit a management or data type frame in the corresponding access phase. An illustrating example is shown in Figure 8. Nodes can be in sleep mode during beacon transmission time, RAP and MAP. A node is in inactive state during its scheduled allocation intervals and polled allocation intervals on determining that the remaining interval has been relinquished by itself or reclaimed by its hub due to no more pending transmissions. If there is heavy traffic for nodes to transmit in the network, they will be definitely busy throughout the superframe without inactive state.

#### 3.2.2. Transmission Progress Description

**Figure 9:**
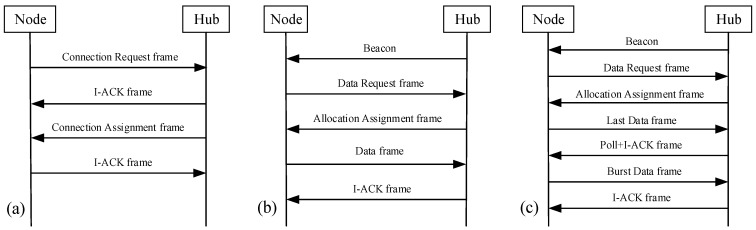
(**a**) Node connection procedure; (**b**) Periodic traffic transfer model; (**c**) Burst traffic transfer model.

An unconnected node shall send a Connection Request frame to a hub and hub send a Connection Assignment frame to the node for the node to be connected with the hub, as presented in Figure 9a. Then, the periodic traffic and bust traffic transfer model is illustrated in Figure 9b,c. For normal periodically data, hub interacts with node twice for successful data transmissions. If there are burst data, more time intercommunication will generate. Hub sends a control frame I-ACK+Poll for burst data polled allocation.

**Figure 10:**
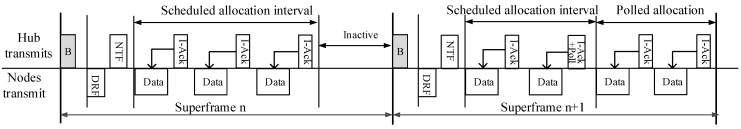
Frame transactions in scheduled and polled allocation intervals.

**Figure 11:**
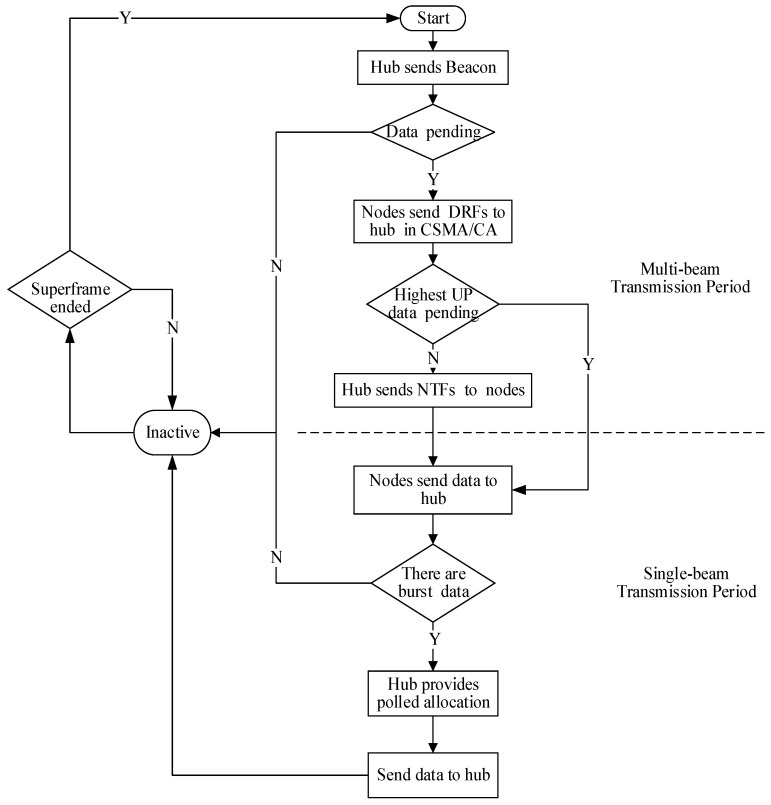
Flowchart of LEDA MAC protocol.

Considering Figure 10, the further frame transactions in LEDA MAC are illustrated. Hub initiates the communication firstly, sending beacons to all nodes to notify control information. If there is highest UP data, the first time slot should be allocated for it. Then the DRFs from nodes contend to access the channel in CSMA/CA in multi-beam mode. The CSMA/CA mechanism with UP considered guarantees the QoS of traffic in higher priority. After NTFs have been received, all nodes turn to single-beam mode. Hub polls nodes in scheduled mechanism for data interactive. The sequence of nodes to send data is regulated in NTFs, mainly according to the traffic designation in Table 1. One node would not initiate a transmission outside of its allocation intervals. Every node knows in what time it could send or receive data. Both the hub and node can point towards each other before data transmission begins. Nodes with burst traffic will process the traffic in polled allocation. An immediate or future polled allocation will be decided by the hub. Configuration of specific fields is illustrated in Figure 7. The nodes with no data transmission will turn into inactive state to save energy. The flow chart is shown in Figure 11.

### 3.3. Deafness and Hidden Terminal Problem Avoidance

**Figure 12:**
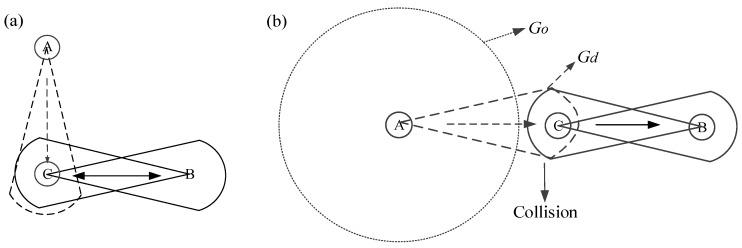
(**a**) A scenario to illustrate the deafness problem; (**b**) A scenario of hidden terminal due to asymmetry in gain.

Deafness and hidden terminal problems are the critical challenges in directional antennas. A scenario to illustrate the deafness problem is shown in Figure 12a. Node C appears deafness to the transmission of node A and cannot respond when it is in directional communication with node B. If B has multiple packets to send to C, A would be engaged in a long backoff period and retransmits packets so many times before it acquires a chance to communicate with node C. The unnecessary retransmissions increase the packet delay and power consumption, reducing the network throughput. Deafness also leads to temporary unfairness between nodes that share a same receiver.

The hidden terminal problem occurs when two nodes that are out of each other’s carrier sensing range attempt to transmit data to a common receiver, causing collision as a result. In directional antennas, the hidden terminal problem appears when a potential interfering node cannot receive the request/notification packets due to its orientation. It is important to note that the antenna gain in directional mode (*G_d_*) is greater than in omni-mode (*G_o_*). A scenario of hidden terminal in Figure 12b happens due to asymmetry in gain. Node A and node B are out of each other’s transmission range when only one of them transmits data in directional mode with gain *G_d_*. However, they are within each other’s range when both of them are in directional-mode, causing a collision at node C.

In LEDA MAC protocol, the deafness and hidden terminal problem can be avoided. In MTP, hub and node interoperate request/notification frames in multi-beam mode that all nodes can hear the packets. Thus the deafness problem can be avoided. In STP, all nodes transmit data to hub in sequence according to the NTFs. Even for burst data, the polled allocation is assigned by hub through control frame I-ACK + Poll. As regards hidden terminal problem, the single-beam gain is symmetrical, *i.e.*, the same with the multi-beam gain in the data transmission period in our model. Moreover, in one-hop wireless body area networks, nodes only exchange data packets with hub and any concurrent transmissions in addition to nodes and hub are not expected to initiate. Thereby, the hidden terminal problem will not happen. The LEDA MAC conquers the deafness and hidden terminal problem, and can reduce the interference of neighbor nodes in single-beam transmission. Meanwhile, data transmissions in one active beam avoid some unnecessary transmissions causing collisions and save energy than that of omni-directional communications.

## 4. Theoretical Analysis

The performance of LEDA MAC is evaluated in this section, focusing on the network throughput, average packet delay, energy consumption and network lifetime. The analysis is conducted under the ideal transmission condition that the packet error occurs only by the bad channel conditions. The communication of medical implanted devices, which considers the human body as an inhomogeneous medium consisting of multiple tissue layers with different electrical characteristic, is not studied here. We only consider the on body radio propagation. According to reference [2,43,44], the propagated waves are more likely to diffract around human body rather than penetrate it. Thus, the traditional wireless communication formulas can be applied. The Friis Equation, as illustrated in Equations (1) and (2) can be used to calculate the path loss. We evaluate the proposed LEDA MAC performance in both unsaturated and saturated network condition.

### 4.1. Throughput

We define the network throughput *S* as the average successful transmitted payloads in per unit time. *T* is the duration of a superframe. In unsaturated network, the throughput changes with the arrival rate of data. Then, the network turns into saturated when arrived data beyond the maximum channel service capacity. Let *λ* be the data arrival rate and *μ* be the deterministic service rate. Nodes with different UPs have the same arrival rate and service rate. The parameter *n* denotes the number of sensor nodes that have data to be transmitted in the network. The network throughput of LEDA MAC in unsaturated and saturated network can be obtained from

(3) $$S\text{=}\left\{ \begin{array}{l} \frac{n^{2}\left(n\lambda T_{aslot}+T_{MTP}\right)}{T}，n\lambda <\mu \\ \begin{array}{ccc} \frac{\mu \left(T_{STP}+T_{inactive}\right)}{T} & ，n\lambda \geq \mu & \end{array} \end{array}\right.$$

where *T_aslot_* is the length of one allocated interval. *T_MTP_* and *T_STP_* are the duration of MTP and STP, respectively. In saturated network, there are always data waiting to be transmitted. The length of DPAP reaches the maximum, the value of which is the length of *T_inactive_* Correspondingly, *T_STP_* reaches its maximum. In addition, *η* is the link utilization ratio that can be calculated by

(4) $$\eta =\frac{T_{data}}{T_{data}+2T_{turn}+T_{I-ACK}+GT}$$

where turnaround time *T_turn_* is the setup time from receive-to-transmit or transmit-to-receive. *GT* is the guard time in scheduled allocation providing fit allocation interval and can be calculated as

(5) GT=pSIFS+pExtraIFS+mClockResolution 

The parameter *GT* comprises the interframe space *pSIFS* between uninterrupted successive transmissions, the synchronization error tolerance *pExtraIFS* and the timing uncertainty *mClockResolution*. *T_data_* is the transmission time of the Physical Layer Protocol Data Unit (PPDU). Considering Figure 3, it is given by

(6) $$T_{data}=\frac{N_{packet}}{R}\;=\frac{N_{preamble}+N_{header}+N_{PSDU}}{R}\;$$

where *R* is the information rate. *N_preamble_* and *N_header_* are the length of PLCP preamble and header, respectively. The number of bits in PSDU is obtained from

(7) $$N_{PSDU}=8\times (N_{MACheader}+N_{MACFrameBody}+N_{FCS})$$

### 4.2. Delay

Considering Figure 5, only request and notification frames are transmitted in MTP of LEDA MAC. Data transmissions occur in STP and DPAP. The average packet delay of LEDA MAC protocol *D_avg_* including the data waiting delay, data transmission delay, the turnaround time consumed and appropriate guard time. Propagation delay of packets is neglected in this paper. *GT* and *T_data_* are calculated in Equations (5) and (6), respectively. Then, *D_avg_* is given by

(8) $$D_{avg}=T_{data}+T_{wait}+2T_{turn}+GT$$

According to the traditional queuing theory on scheduled mechanism, the mean waiting delay. *T_wait_* in a queue can be calculated by

(9) $$T_{wait}\text{=}\left\{ \begin{array}{l} \frac{n\lambda T_{aslot}}{2(\mu -\lambda )}+\frac{T_{aslot}}{2}+\frac{\sum_{i=1}^{n}\left(n-i\right)T_{aslot}}{n}+\frac{T_{MTP}}{2}，n\lambda <\mu \\ \begin{array}{ccc} T_{MTP}+\frac{\mu }{n\lambda }\left(\sum_{i=1}^{n}\frac{(n-i)T_{aslot}}{n}+\frac{T_{aslot}}{2}\right)+\frac{n\lambda -\mu }{n\lambda }\times T & , & \end{array}n\lambda \geq \mu \end{array}\right.$$

where *T_MTP_* is the time cost by the management frames including data request frames and notification frames in request and notification period. It is obtained by

(10) $$T_{MTP}=\bar{T_{DRF}}+T_{NTF}=T_{cw}+T_{DRF}+T_{NTF}$$

where $\bar{T_{DRF}}$ is the average consumed time of a successful transmitted data request frame in request period, including the average backoff time of data request frames *T_cw_* and the transmission time *T_DRF_*. Once the data transmission is successful, the CW is set to *CW_min_*. Always the *T_cw_* of successive transmission can be obtained as

(11) $$T_{cw}\text{=}\frac{CW_{\min}[UP_{k}]\times \left(T_{CCA}+T_{pMIFS}\right)}{2}$$

where *T_CCA_* is the time needed to execute Clear Channel Assessment (CCA) mechanism in CSMA/CA. *pMIFS* is the minimum interframe separation time with the stationary value 20 μs. Interframe separation time is defined as time elapsed from when the last sample of the last transmitted symbol is present on the air interface, to the time when the first sample of the first transmitted symbol of the PLCP preamble for the following packet is present on the air interface.

### 4.3. Energy Consumption

Nodes in the WBAN switch between active and inactive state. The average energy consumption in LEDA MAC *E_LEDA_* can be obtained from the following expressions.

(12) $$E_{LEDA}\text{=}E_{active}+E_{inactive}$$

(13) $$E_{active}\text{=}E_{rx-m}+E_{tx-m}+E_{rx-s}+E_{tx-s}+E_{oh}$$

where $E_{tx-m}$ and $E_{rx-m}$ are the energy consumed in transmit and receive states in multi-beam mode. The corresponding energy consumption can be calculated as Equation (14).

(14) $$\begin{array}{l} E_{rx-m}=P_{rx-m}\times T_{rx-m}=P_{rx-m}\times \left(T_{Beacon}+T_{NTF}\right)\\ E_{tx-m}=P_{tx-m}\times T_{tx-m}=P_{tx-m}\times \bar{T_{DRF}}\times n \end{array}$$

$E_{tx-s}$and $E_{rx-s}$ represent the energy consumed when the nodes transmit or receive data in single-beam mode. Define *k* as the number of data packets arrived of one node in a superframe period *T*, the value of which is $k=\frac{\lambda T}{N_{packet}}$. In unsaturated network, all arrived data can be transmitted successfully, and in saturated network, the maximum network service rate is μ. As a result, the energy consumed in single-beam mode can be given by

(15) $$E_{rx-s}=P_{rx-s}\times T_{rx-s}=\left\{ \begin{array}{l} P_{rx-s}\times T_{I-ACK}\times nk\;,n\lambda <\mu \\ P_{rx-s}\times T_{I-ACK}\times \frac{\mu k}{\lambda }\;,n\lambda \geq \mu \end{array}\right.$$

(16) $$E_{tx-s}\text{=}P_{tx-s}\times T_{tx-s}=\left\{ \begin{array}{l} P_{tx-s}\times T_{data}\times nk\;,n\lambda <\mu \\ P_{tx-s}\times T_{data}\times \frac{\mu k}{\lambda }\;,n\lambda \geq \mu \end{array}\right.$$

Energy consumed due to overhead includes antenna turnaround power, wake up power (the power consumed when device moves from inactive to transmit/receive state). The overhead energy consumption *E_oh_* can be obtained using Equation (17).

(17) $$E_{oh}=E_{turn}+E_{wakeup}=n\times (P_{turn}\times 2T_{turn}\times k+P_{wakeup}\times T_{wakeup})$$

Nodes turn from active state into inactive state to save energy if there is no data pending for transmission. Thus the energy consumption in inactive mode or sleep mode *E_inactive_* in unsaturated network is

(18) $$E_{inactive}=P_{inactive}\times \left[T-T_{rx-m}-T_{tx-m}-T_{rx-s}-T_{tx-s}-nk\times \left(\text{2}T_{turn}+T_{wakeup}\text{+}GT\right)\right],\;n\lambda <\mu$$

where the $E_{inactive}=0,\;n\lambda <\mu$ in saturated network because nodes will send data continuously to hub without inactive period due to the heavy data traffic. Under this circumstance, the data transmission will last the whole superframe period, and costs relatively more energy.

### 4.4. Network Lifetime

Sensor nodes are expected to live as long as possible due to the constraints of battery capacity. The network lifetime is designed as time duration from the start of network operation to the moment a node exhausts its energy. The value of network lifetime is equal to the shortest lifetime of a sensor node. According to the theoretical analysis of energy consumption above, we can evaluate the lifetime of sensor nodes. Here we assume all the nodes are in the same configuration. Every node applies a button cell of capacity Q mAh as energy supply. Supposing the average energy consumption of a sensor node in a superframe is *E_node_*, the survival time of sensor nodes can be calculated from

(19) $$T_{suv}=\frac{Q}{E_{node}}\times T$$

where *T* is the duration of superframe. Considering Equations (12)–(18), the *E_LEDA_* is the average consumption of all the nodes in the network. Thus the *E_node_* can be given by

(20) $$E_{node}=\frac{E_{LEDA}}{n}$$

## 5. Simulation Results

In this section, we present numerical analytical and simulation results of the LEDA MAC performance evaluation. We have made a comparison of LEDA MAC protocol and IEEE 802.15.6 MAC protocol on MIRAI-SF (MIRAI Simulation Framework) platform. The MIRAI-SF is a scalable simulation framework based on discrete event simulation technology. The relevant parameters used in the simulation are listed in Table 2. Typical power consumption data for sensor nodes are derived from the well-known CC2420 chip [45].

**Table 2:** Simulation parameters.

Parameters	Value	Parameters	Value
Information rate	971.4 kbps	Beacon	15 bytes
Superframe length	1 s	PLCP preamble	90 bits
Slot length	5 ms	PLCP header	31bits
Transmit power	−3 dBm	MAC header	7 bytes
Received power	0 dBm	Wakeup	8 bytes
Directional transmit power	−25 dBm	MAC ACK size	9 bytes
Directional received power	−22 dBm	Payload	255 bytes
Inactive power	4 μw	Buffer size	20,000 bytes
Turnaround time	75 μs	Simulation time	30 s
pSIFS	75 μw	Frequency band	2400–2438.5 MHz

In this paper, one-hop star topology structure is employed in the simulation scenario. Each node in the network produces one UP traffic. The simulation is conducted under the ideal transmission condition. The effect of bit errors in the channel is neglected. In other words, a packet is dropped only due to packet collision or device buffer overflow.

**Figure 13:**
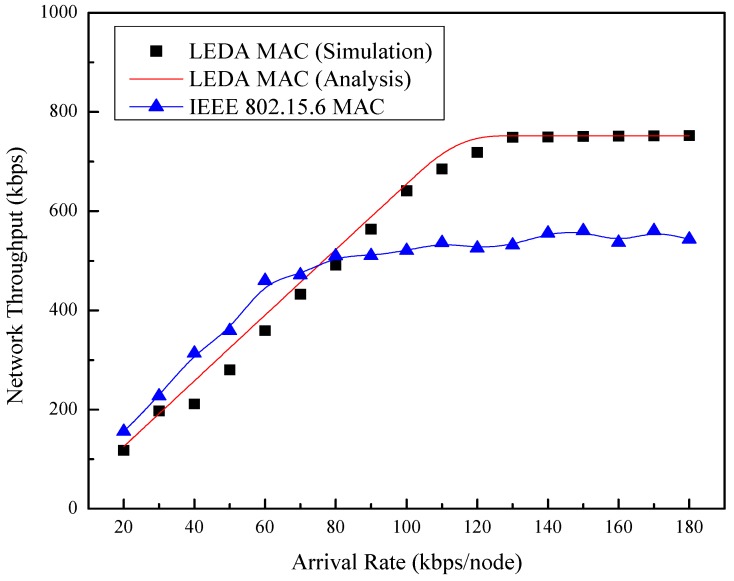
Network throughput *vs.* arrival rate.

The relationship between network throughput and the arrival rate is shown in Figure 13. In this simulation, one hub and eight nodes are included. Network throughput of the two protocols increases as the arrival rate increases. Changes are smaller before the network traffic is saturated. The IEEE 802.15.6 MAC shows higher throughput than LEDA MAC owing to the data transmission in RAP before reaching its maximum throughput. However, the maximum throughput of LEDA MAC protocol increases almost 60% than that of IEEE 802.15.6, which is attributed to the dynamic allocation scheme. Thus, the better performance of LEDA MAC protocol can be obtained in high traffic condition.

**Figure 14:**
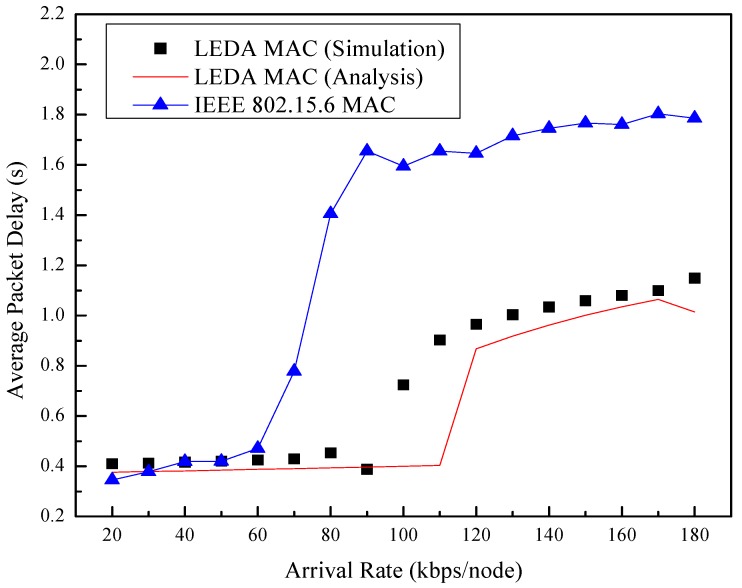
Average packet delay *vs.* arrival rate.

Figure 14 depicts the average packet delay of the network *versus* the arrival rate. Burst data can be sent in the current superframe in LEDA MAC protocol instead of waiting for the next superframe in unsaturated network. So the packet delay of LEDA MAC is far lower than that of IEEE 802.15.6. Moreover, the orientation property of directional antenna reduces the collision possibility and interference of neighbor nodes, reducing the packet delay. Here, we employ the maximum information rate in WBAN, the value of which is 971.4 kbps. There are eight nodes in the network, the arrival rate of which is the same. When the arrival rates of the network beyond the network service rate, the network turns into saturated state. Accordingly, the access delay will suffer a sharp increase. It is obviously that the analytical transition point of LEDA MAC appears between the arrival rate 110 kbps and 120 kbps of each node. The simulated results of LEDA MAC show better adaptability than that of IEEE 802.15.6 MAC, which contributes to the dynamic polled allocation mechanism. The sharp increase of the delay curves appears since arrived data have to wait for the next superframe to be transmitted due to heavy traffic beyond the service capacity. When the network traffic is saturated, newly arrived data are stored in the buffer. Therefore, the packet delay changes little for caching data.

**Figure 15:**
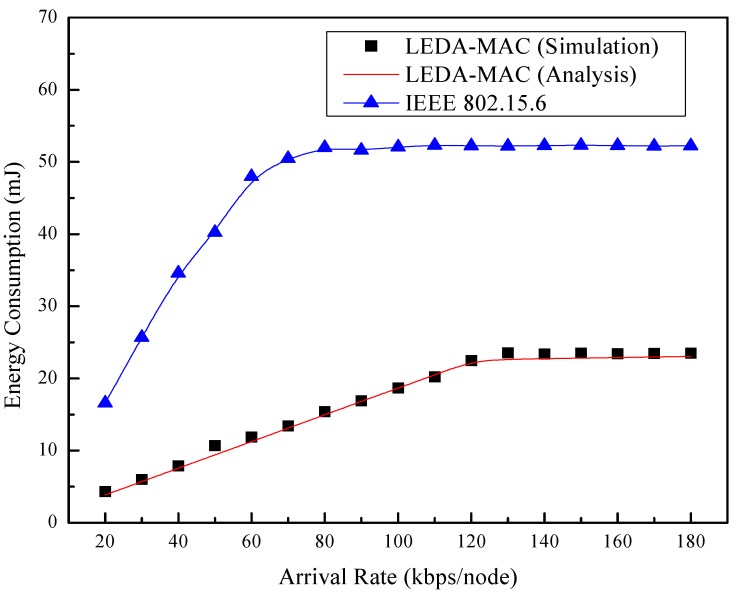
Total energy consumption *vs.* arrival rate.

The changes of total energy consumption with *versus* arrival rate are presented in Figure 15. The total energy consumption is the summation of energy consumed of all nodes including the hub and ordinary nodes. LEDA MAC saves the energy consumption significantly than that of IEEE 802.15.6 MAC both in saturated and unsaturated network owing to the directional antennas technology. IEEE 802.15.6 consumes more than twice the energy of our proposed directional MAC. Furthermore, the analytical and simulation results of LEDA MAC match well.

**Figure 16:**
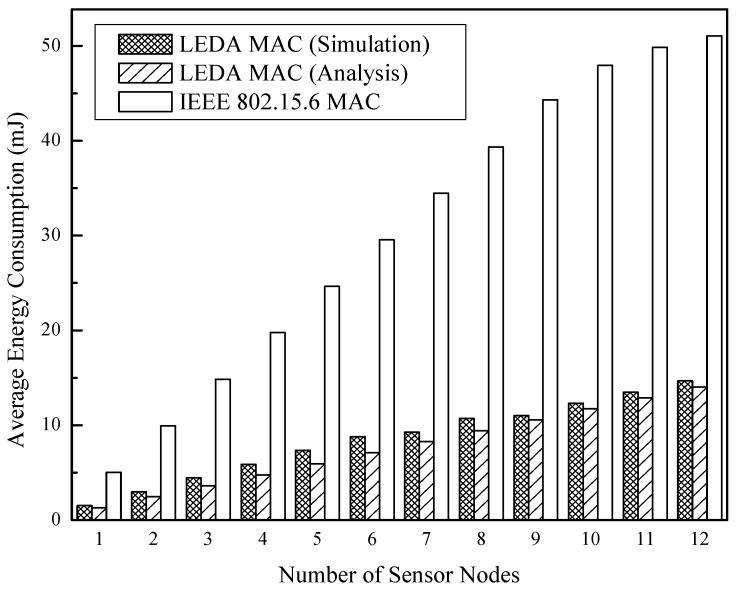
Total energy consumption at 50 kbps/node *vs.* number of sensor nodes.

**Figure 17:**
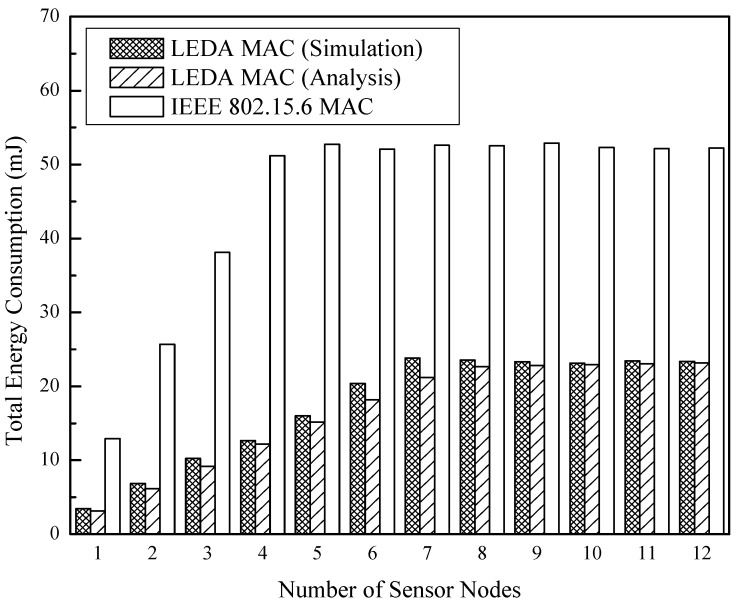
Total energy consumption at 130 kbps/node *vs.* number of sensor nodes.

Besides, the relationship between energy consumption and the number of sensor nodes is obtained in Figure 16 and Figure 17. Both of the two MAC protocols are in unsaturated at the arrival rate of 50 kbps per node, but IEEE 802.15.6 MAC consumes 70% additional energy than LEDA MAC. The network changes from unsaturated state to saturated state with the increasing number of sensor nodes with data arrival rate 130 kbps. Both of the two protocols consume much energy due to heavy data transmissions, as indicated in Figure 17. Whereas, at an equivalent data arrival rate, the LEDA MAC saves 56% energy and can adjust to more nodes in high throughput condition than IEEE 802.15.6.

**Figure 18:**
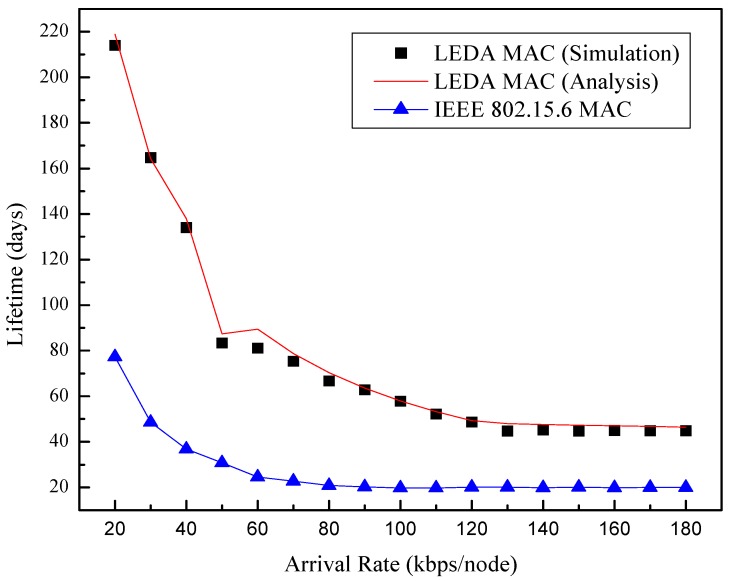
Lifetime of network *vs*. arrival rate.

We evaluate the network lifetime with the change of data arrival rates in the assumption that the eight nodes are equipped with a button cell, the capacity of which is 560 mAh. Considering Figure 18, it is apparent that the LEDA MAC can save more energy than IEEE 802.15.6 with the maximum lifetime of 214 d while IEEE 802.15.6 is 77 d. Even in heavy traffic condition, the network lifetime of LEDA MAC is twice longer than that of IEEE 802.15.6. The results further confirm the directional mechanism is effective in energy saving. The single-beam directional mode of LEDA MAC for data transmission can significantly extend the network lifetime, which is appropriate for health monitoring in WBAN.

**Figure 19:**
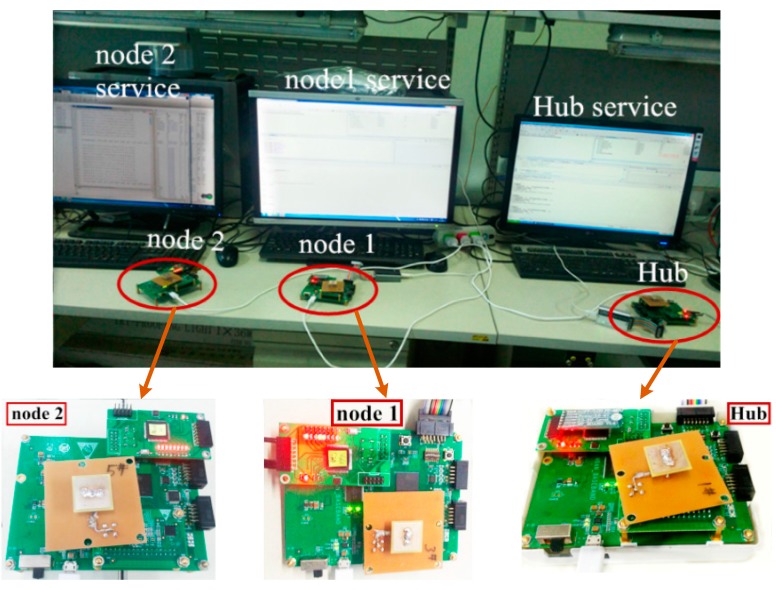
An experiment scenario of WBAN with three nodes.

More than the aforementioned simulation results, we implement the MAC protocols in experimental scenario. A minimum WBAN in our independent research and development with one hub and two sensor nodes is shown in Figure 19. We have realized both CSMA/CA and scheduled mechanisms for data transmissions in WBAN. The tentative study of the performance of IEEE 802.15.6 MAC has been implemented on it in laboratory experiment scenario. An experimental scenario is illustrated as follows. Thousands of frames with the length of 30 bytes are transmitted to evaluate the performance. The minimum WBAN transmits the frames at the information rate 121.4 kbps while each node keep a 30 cm distance with the hub. The experimental results show the normalized throughput ranges from 40.77% to 50.63%. Meanwhile, the Frame Error Rate (FER) is less than 3.8%. Whereas, the peer to peer transmission can obtain a high performance. The FER is 0.005 at the 1 m distance. Even the distance up to 2 m, the FER remain unchanged. The original intention is to demonstrate and share the significance of our work. We know profoundly the experimental results are interim achievements that need to be further improved.

## 6. Conclusions

The burning problem of how to minimize energy consumption of sensor nodes to prolong network lifetime in WBAN is in the spotlight of attention for both industry and academia. This paper proposes a hybrid lifetime extended MAC protocol with directional antennas named LEDA MAC to decrease energy consumption and extend network lifetime. Normal periodically data and burst data employ two different directional reservation and allocation mechanisms. Both analysis and simulation results demonstrate the energy consumption of LEDA MAC can be reduced by more than 56% of IEEE 802.15.6. Simultaneously, the nodes’ lifetime is 176% higher compared to IEEE 802.15.6, which is beneficial to health monitoring. Optimizing power control of LEDA MAC protocol to achieve optimum results is our future work. Moreover, we will execute the experiment of both IEEE 802.15.6 and LEDA MAC in a larger-scale WBAN with more sensor nodes added. Furthermore, the implementation of our proposed protocol on a platform equipped on a human body in an actual scenario is scheduled.
